# Sacrificial gold coating enhances transport of liquid metal in pressurized fountain pen lithography

**DOI:** 10.1038/s41598-021-84065-4

**Published:** 2021-02-25

**Authors:** Gideon I. Livshits, Jiannan Bao, Leo Sakamoto, Tomoki Misaka, Yuki Usami, Yoichi Otsuka, Takuya Matsumoto

**Affiliations:** 1 Department of Chemistry, Graduate School of Science, Osaka University, 1-1 Machikaneyama-cho, Toyonaka, Osaka 560-0043 Japan; 2 Present Address: Lev Academic Center, Jerusalem College of Technology, Havaad HaLeumi St. 21, Jerusalem, 9116001 Israel

**Keywords:** Wetting, Design, synthesis and processing, Materials science

## Abstract

Liquid metals have attracted attention as functional components for moldable electronics, such as soft flexible connectors, wires or conductive ink. The relatively high surface tension (> 400 mN m^−1^) and the fact that liquid metals do not readily wet ceramic or oxide surfaces have led to devising unique techniques to spread the liquid and mold its shape. These techniques include surface modification, electrowetting and vacuum filling of channels. This work presents an injection technique based on pressurized fountain pen lithography with glass nanopipettes developed to directly pattern liquid metal on flat hard substrates. The liquid metals were eutectic alloys of Gallium, including Gallium-Indium (EGaIn), Gallium-Indium-Zinc and Gallium-Indium-Tin. The nanopipettes were coated internally with gold, acting as a sacrificial layer and facilitating the wetting of the pipette down to its pore, with an inner diameter of ~ 100–300 nm. By applying hydrodynamic pressure to the connected end of the pipette, the metal was extruded through the pore, forming long continuous (> 3 mm) and narrow (~ 1–15 µm) metal lines on silicon oxide and gold surfaces at room temperature and ambient conditions. With this robust platform, it is possible to pattern liquid metals on a variety of substrates and geometries down to the micron range.

## Introduction

Various methodologies of direct writing of liquid metal shapes and functional components have been described in the scientific literature^1–10^. In particular, Ladd et al*.*^5^ demonstrated 3D printing of free-standing liquid metal microstructures of varying sizes, and Yoon et al*.*^2^ demonstrated a platform for direct writing of liquid metal patterns, 70–80 µm wide, on uneven or curved surfaces. The interest in such techniques lies in their top-down control over architecture, enabling the formation of 2D or 3D electrically conductive structures without the need for sacrificial masking steps, which are routinely employed in standard lithographic techniques. The main challenge in developing a reproducible and controllable platform for this type of lithography is the transition from the macro-scale to the micro or nano regime due to the native high surface tension of liquid metals^11, 12^, which makes it very difficult to form well-defined micro/nano-structures. Systematic improvements in pattern resolution were made by Boley et al*.*^1^ who demonstrated writing lines with various widths from 535 µm down to 83 µm, and more recently, by Shin et al.^13^, who reported printing lines with a width as small as 22 µm on flat, inclined and curved substrates. In the latter study, the improved resolution was mainly due to sensor-control of the distance between the substrate and the tip of the nozzle in the vertical direction. This enabled the authors to utilize metallic nozzles with an inner diameter that was 3–5 times the width of the drawn line. Park et al.^9^ have taken this technique a step further, by utilizing glass pipettes instead of metallic nozzles, with inner diameters ranging from 5 to 40 µm. By controlling the tip-substrate distance, they were able to obtain controlled pattern features with a minimal width of just under 2 µm. In the present work, we address this challenge further by proposing to utilize glass nanopipettes to control the pore size down to the 0.1 µm scale.

**Figure 1:**
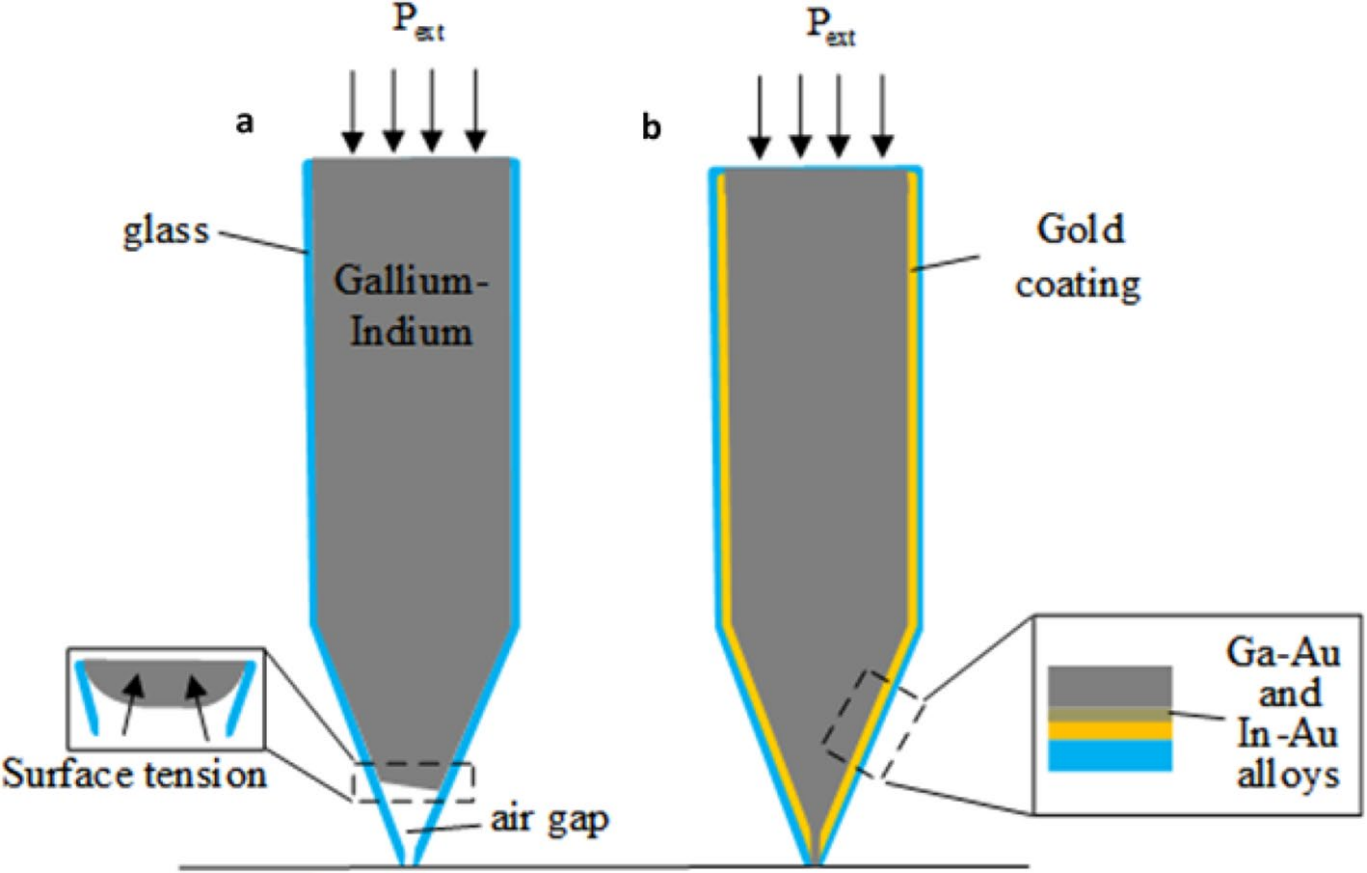
Experimental Scheme. (**a**) When external pressure is applied to the liquid EGaIn inside the glass pipette, it proves insufficient in overcoming the surface tension, leaving a gap of air, beyond which it would not fill. Continuing to apply the pressure beyond this point typically results in the explosive shattering of the glass pipette. (**b**) When the glass is coated with gold from the inside, the molecular interactions between the gold coating and the liquid metal enable wetting under pressure. As the pressure increases, the liquid fills up to the pore, while the gold layer is absorbed almost immediately into the metal liquid.

Working with open-ended nanopipettes requires much higher pressures to overcome the surface tension of the liquid material inside microscale apertures^9^. When properly configured, as we shall demonstrate in this manuscript, the resulting patterns can be reduced to a width of 1 µm. In order to drive the metal liquid into the nanopipette, we experimented initially with several methodologies, such as electrowetting using an adjacent counter-electrode^14–16^ and heating-induced spreading and wetting using as a heating element a coil wrapped around the pipette^17^. These techniques showed limited promise and proved too weak (even at ~ 10 kV and ~ 400 °C, respectively) to overcome the glass-metal wetting barrier to manipulate EGaIn through the sub-micron aperture of the pipette. To address this challenge, we opted instead for chemical or reactive wetting^12^, by exploiting the affinity of both Gallium and Indium to form metal alloying compounds with gold^18–22^. A schematic representation of the experiment is shown in Fig. 1. When the metal liquid is introduced into a pristine uncoated glass pipette (Fig. 1a), it forms a boundary due to its surface tension, and an air gap is formed between this boundary and the pore. It is possible to apply pressure to the liquid metal to force it farther into the tube, but at a certain maximum pressure the tube would shatter. In order to reduce the needed pressure and facilitate the filling process by effectively breaking the boundary, we induce wetting at the glass walls by coating the inner surface of the glass capillary with gold (Fig. 1b). It is the alloying reactions around the inner surface that break the boundary or at least make it unstable enough, so that the thin gold layer is consumed by EGaIn, and the pressure that is required to fill the tube is significantly reduced.

## Results

**Figure 2:**
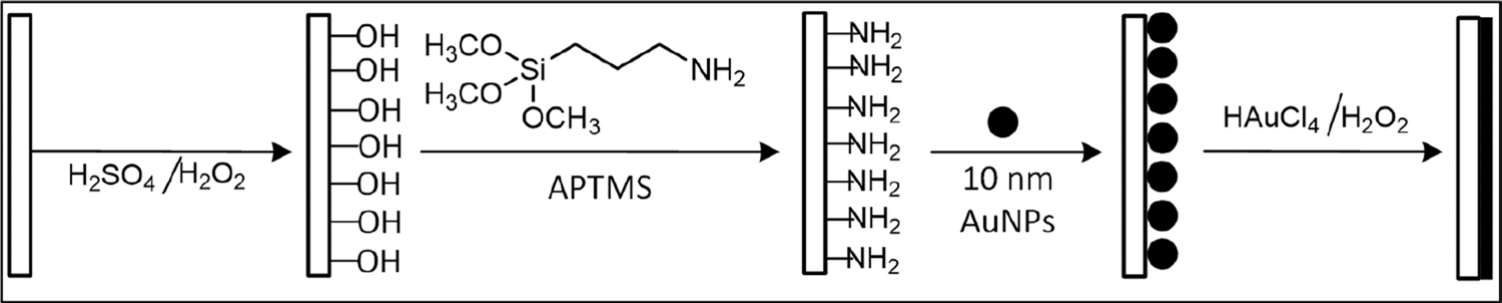
Scheme of the chemistry of the gold coating of glass. The illustration is adapted from Hu et al.^23^ Left to right: after cleaning and surface activation, the inner surface of the glass tubes was functionalized with a solution of APTMS overnight by pumping the solution directly through the tubes. After thoroughly cleaning the tubes from the inside, they were seeded with 10 nm GNPs for 3–4 h, and electroless deposition was activated by introducing a solution of Gold (III) chloride trihydrate and H_2_O_2_ as a reducing agent. The reaction commenced as soon as the solution reached the cavity and was allowed to continue for 20 min. The procedure is demonstrated in Fig. S1 with more details in the “Methods” section.

**Figure 3:**
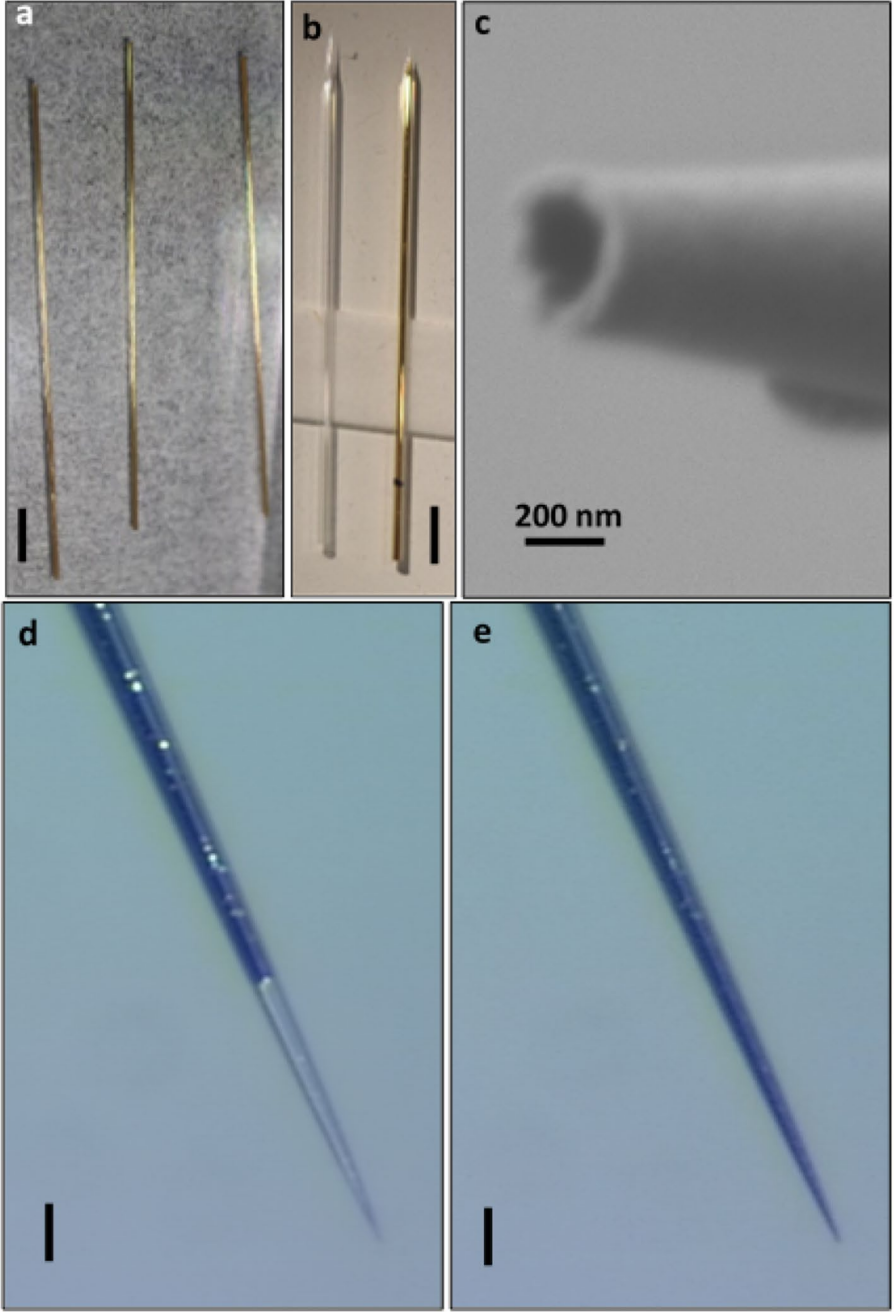
Pipette coating and filling. (**a**) Optical image of three gold-coated glass capillaries. The coating is only from the inside; it is continuous and conductive. Scale bar is 10 mm. (**b**) Optical image of two nanopipettes, bare (left) and coated (right), for comparison. The coating becomes sparser towards the aperture due to the thermal extrusion process. Scale bar is 5 mm. (**c**) SEM micrograph of a tip of a coated pipette as in (**b**), formed under thermal extrusion. (**d**,**e**) Optical images of a coated nanopipette, at the initial, (**d**), and final, (**e**), stages of filling with EGaIn, respectively. Scale bar is 20 µm. Traces of the coating are barely visible, having been dissolved inside the EGaIn. To completely fill the pipette requires ~ 15–25 atm. Once the pipette is filled, the pressure is dropped, and the pipette remains full. A movie of the filling process is available as Supplementary Information S1.

In order to obtain a smooth and uniform coating of the glass nanopipette, prior to its fabrication, the glass capillary was coated with gold from the inside using electroless deposition following published protocols^23, 24^ with several key modifications, depicted schematically in Fig. 2. Most notably, surface functionalization with (3-Aminopropyl)trimethoxysilane (APTMS), self-assembly of the gold nanoparticles (GNPs) and the subsequent reduction of the gold salt were all done by pumping the solutions directly through the glass capillaries, thereby ensuring the localization of the reaction to the inner surface of the capillaries. Fig. S1 in the Supplementary Information shows the experimental setup with the connected tubes at the final stages of the reaction, as the color inside changes from dark red to gold. A complete description of the chemical synthesis is given in the “Methods” section. The resulting coverage, shown in Fig. 3a, is uniform and adheres well to the glass with an estimated thickness of ~ 100–300 nm.

Nanopipettes were formed from coated capillaries using two different pulling techniques, one by thermal heating, and the other by localized laser heating. Although laser heating can control the formation of the cone shape and the aperture size independently, the resulting tips proved more brittle as they broke down under an applied pressure of ~ 60 atm. In contrast, the thermally-heated nanopipettes could withstand pressures exceeding ~ 100 atm. This behavior may be attributed to non-uniform thermal relaxation of the glass during rapid localized laser heating as compared with the slower radiative and convective heating involved in the thermal coil heater. Therefore, in this study, only thermally-pulled pipettes are presented. From a single coated capillary two nanopipettes were formed by inserting the capillary into a tungsten thermal heater with preset values for all parameters, including the power, weight and heating steps, adjusted to produce a pore size of the inner diameter that is typically 100–300 nm (Fig. 3b,c). See “Methods” section for details. The coated pipette was then filled with EGaIn, and the application of external water pressure resulted in the metal filling the entire volume of the pipette down to its pore (Fig. 3d,e) without clogging by surface tension. A movie of the complete filling process is given in the Supplementary Information.

**Figure 4:**
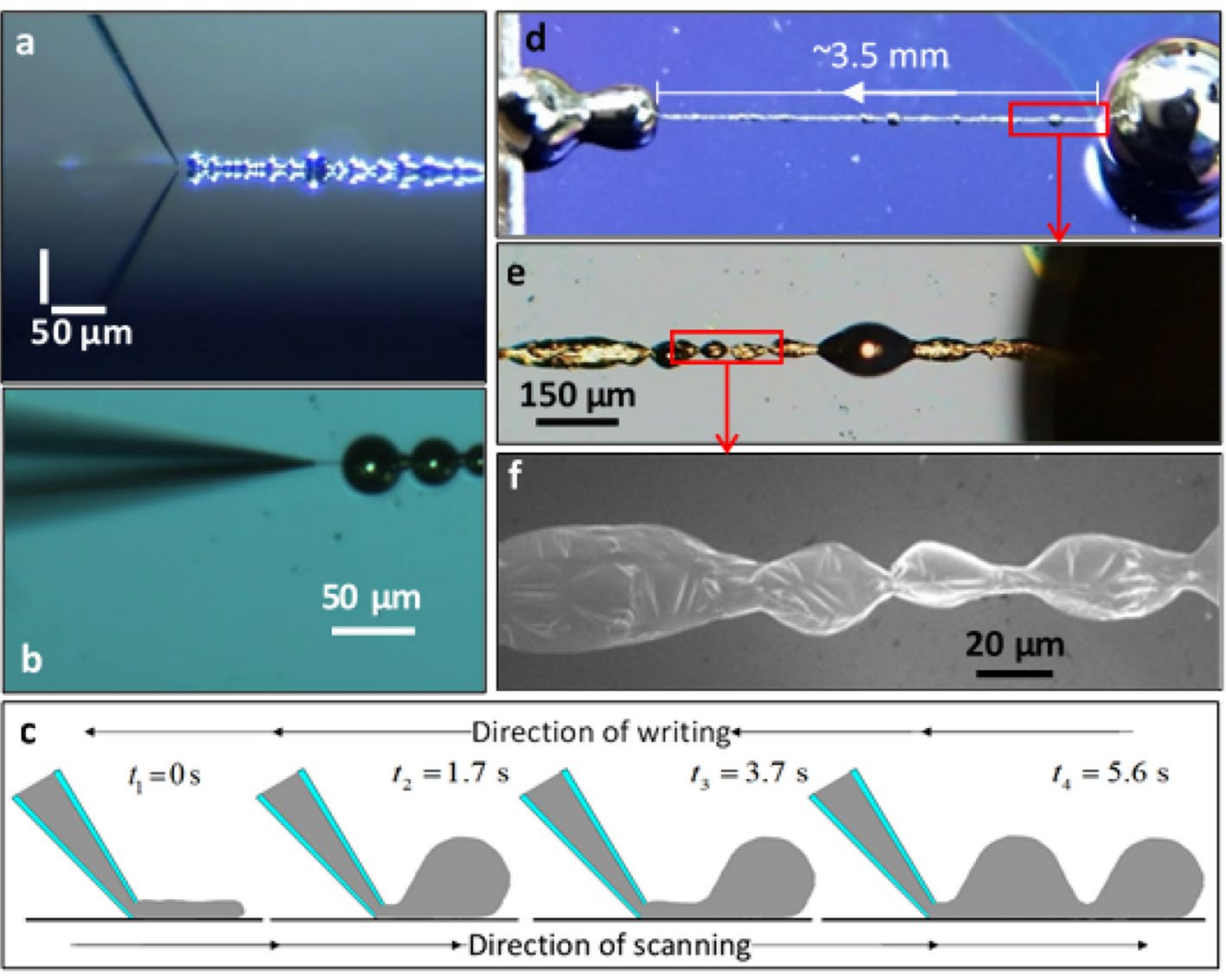
EGaIn on SiO_2_. (**a**,**b**) Optical images (side and top view, respectively) of the process of extrusion (under constant pressure, ~ 45 atm) of EGaIn on a hydroxilated silicon surface. Microdroplets of the EGaln are apparent. (**c**) A schematic illustration of the process (left to right): after a short segment of the line is released onto the surface, typically 15–20 µm long and 1–3 µm wide, the line quickly congeals (after 1.7–1.9 s) into a microdroplet. As the substrate is being moved in the opposite direction, the metal is continuously extruded, a second line is formed, and then again congeals. In this way, a series of joined microdroplets is formed on the surface. See Supplementary Information S2 for a movie of this dynamic deposition. (**d**) Optical image of the completed pattern, extending for over 3.5 mm. (**e**) Optical image of the marked region in (**d**), showing the area near the initial release (right). (**f**) Electron micrograph of the marked region in (**e**), demonstrating in higher resolution the local structure. The droplets are deformed ellipsoids of varying lengths and widths. The initial stage of the line formation is shown in detail in the Supplementary Information, Fig. S2.

**Figure 5:**
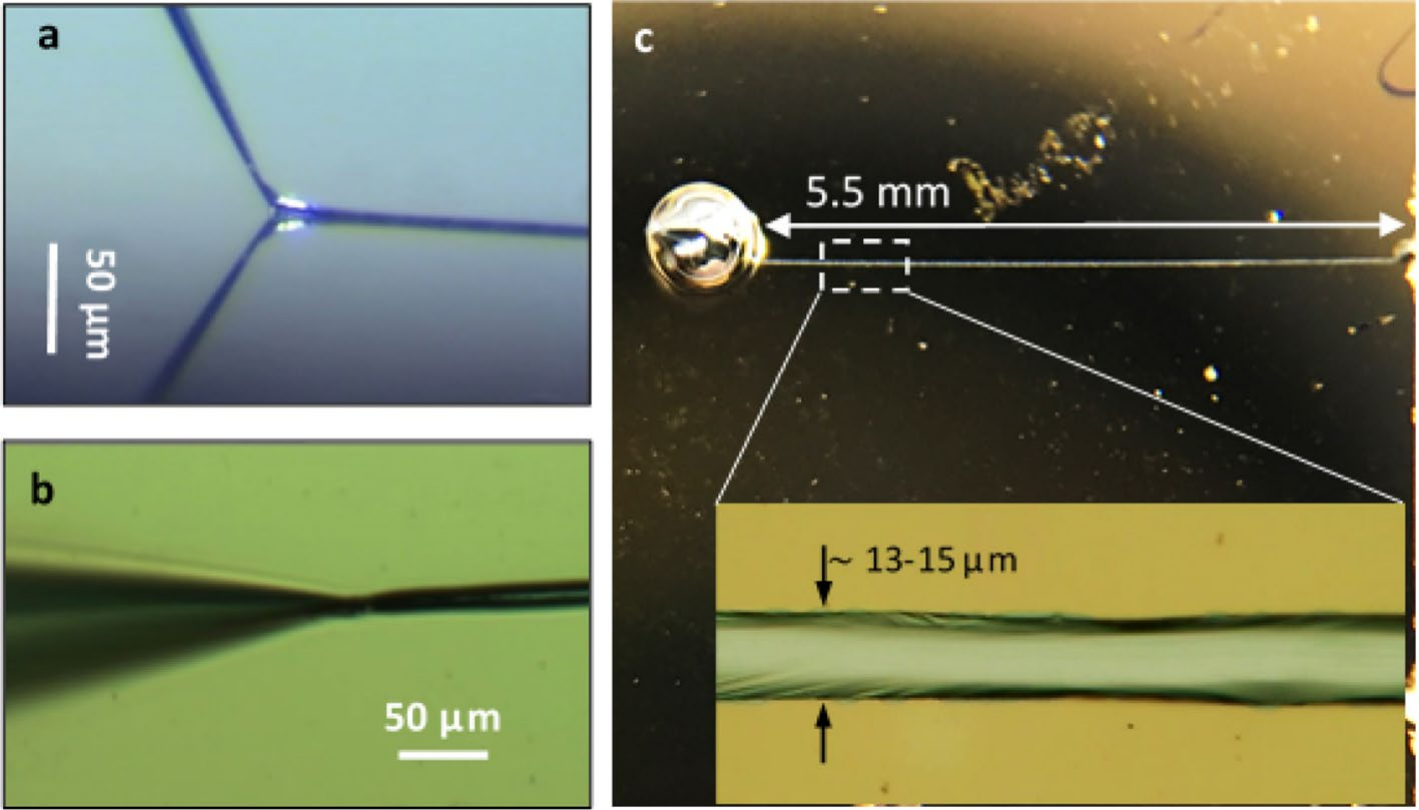
EGaIn on Au. (**a**,**b**) Optical images (side and top view, respectively) of the process of extrusion (under constant pressure, ~ 55 atm) of EGaIn on a gold surface. Once the initial droplet appears, the metal line that emerges is continuous. The entire deposition took ~ 10 min. See Supplementary Information S3 for a complete movie of the deposition. (**c**) Optical image of the completed pattern taken immediately after deposition. The inset is an optical image at higher resolution of the marked area in the main image, taken directly after patterning, showing a very clean deposition with sharp boundaries. Line width is 13–15 µm.

**Figure 6:**
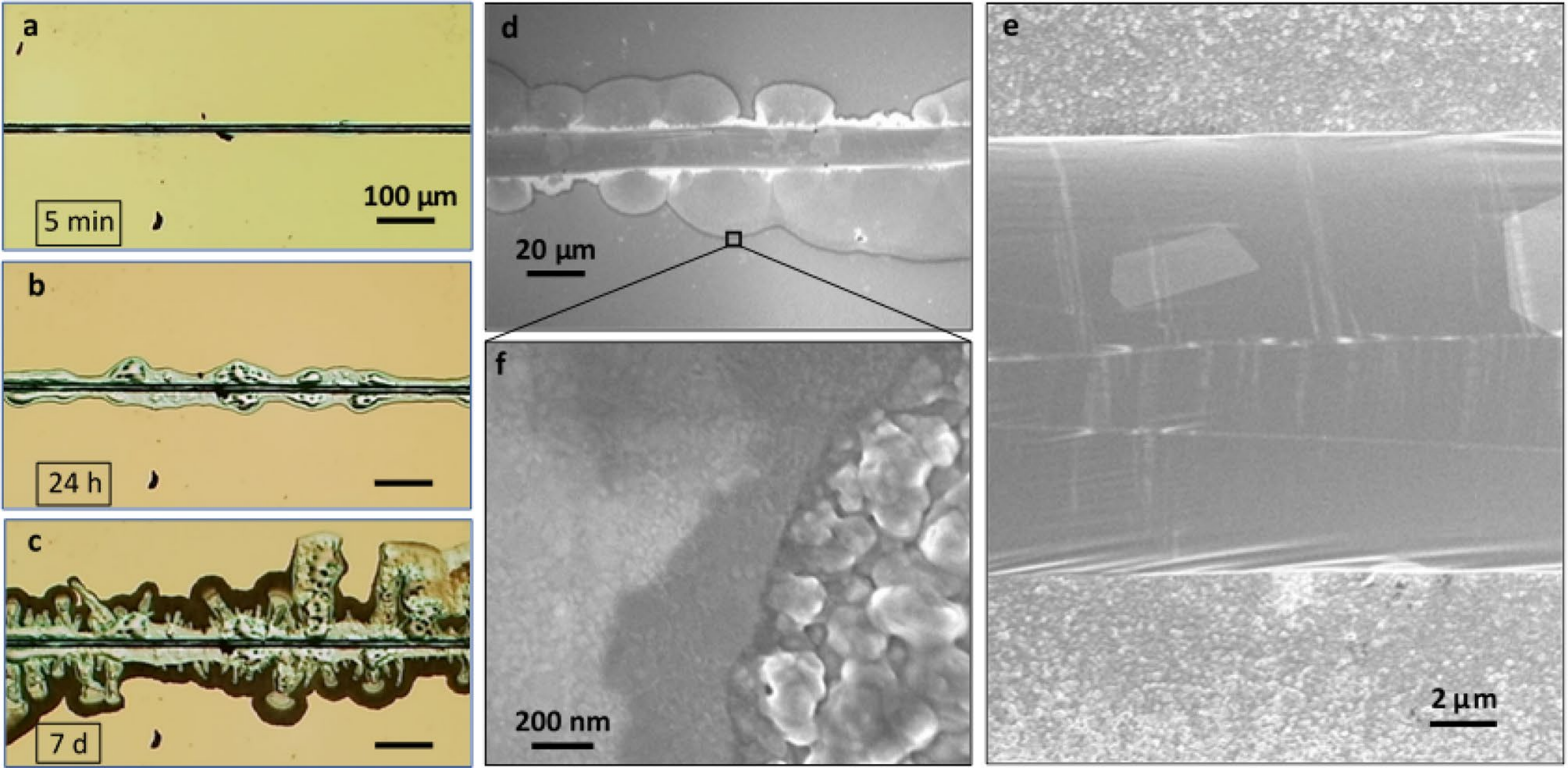
Surface spreading and reactivity. (**a**–**c**) Optical microscopy images of a segment of the patterned line in Fig. 5. The sample was kept inside a clean container at room temperature for several weeks. Image (**a**) was taken immediately after patterning showing a clear line, while (**b**) and (**c**) were taken 24 h and 7 days after patterning, respectively. The gradual spreading of the fronts is clearly visible as Ga and In form alloys with Au. (**d**–**f**) are electron micrographs of the pattern in (**b**). Large crystals are observed inside the line itself (**e**). Higher magnification of the front in (**f**) reveals different crystallite formation, varying in size as a function of the distance from the main source, from larger Ga-rich to smaller ones until the polycrystalline gold coating is reached.

The nanopipette was then brought into contact with the underlying surface, and was kept stationary as the tip was being raster scanned against the moving substrate, at a typical rate of 10 µm s^−1^. Directly after the scanning commenced, pressure was applied to the top of the nanopipette at a constant flow rate, typically between 0.001 and 0.01 ml min^−1^. The buildup of pressure at the pore would increase precipitously until the visible eruption of a liquid metal drop on the surface, typically appearing at the range of 40–60 atm. Immediately following the eruption of the liquid, the external pressure was terminated, but the actual pressure difference would remain steady, dropping only slightly (by about 2–3%). This pressure was observed to be constant, and the scanning would continue uninterrupted, all the while the metal liquid was flowing under this pressure difference with no additional external pressure. The scanning motion enabled the formation of continuous patterns extending for several millimeters (Figs. 4, 5 and 6).

Figure 4 shows the result of drawing EGaIn on a silicon oxide surface. As the metal is extruded through the aperture (Fig. 4a,b), it expands by an order of magnitude, from an inner diameter of roughly 100–300 nm to a width on the surface of 1–3 µm. This printed line is unstable, and when a segment of the extruded metal reaches a length of 15–20 µm (after ~ 1.7 s), the extruded material seems to spontaneously coalesce into a perfect droplet. This is a delayed reaction of the metal to the underlying ceramic surface and to the continual infusion of pressurized metal from the tip. It should be noted that since the pressure gradient is driving the extrusion, it is necessary to choose a scanning rate that would respond to this change and allow a sufficiently quick release of liquid metal. In this respect, it was found that slow rates (*e.g.*, decreasing the rate from 10 to 2 µm s^−1^) produced very large globules of metal that coalesced into even bigger droplets of diameters > 100 µm.

During the translation of the substrate, the metal line continued to form continuously in the opposite direction, and so the formation of the droplets repeated itself. The resulting pattern appeared to consist of connected microdroplets. This process is illustrated in Fig. 4c. Since the lithography set-up is carried out at room temperature in ambient conditions, it is reasonable to assume that a passivation layer of oxide^6, 25^ is formed as the metal is extruded, and that this layer eventually grows to cover the entire network of droplets. Optical and electron imaging (Fig. 4d–f) taken directly after deposition (Fig. 4d) and a day later (Fig. 4e,f and Fig. S2) show more complicated structures. The stable line appears to consist of a continuous network of elongated ellipsoids, with shorter narrow segments (~ 5 µm wide) connecting bigger ones. These deformations of the perfect spherical shape could be attributed to the interplay between the surface tension of the liquid and the elastic properties of the oxide film that covers the liquid, suggesting that the adhesion of EGaIn to the SiO_2_ surface is not strong enough to maintain line shape opposing surface tension. A movie of the direct writing of the line is provided in the Supplementary Information.

To enhance the adhesion of EGaIn to the substrate surface, we capitalized on the observed alloying reaction of EGaIn to Au, and introduced an Au layer on the hydroxylated Si substrate. Figure 5 shows the result of drawing EGaIn on a gold surface, which was prepared by thermal vapor deposition of ~ 100 nm Au on an SiO_2_/Si wafer with Cr as an adhesion layer. In this case, a continuous line is drawn, without any condensation or droplet formation (Fig. 5a–c). The line is markedly wider than the line on the silicon oxide surface, with a width of 10–15 µm (inset in Fig. 5c), possibly the result of the ensuant chemical reactions between the underlying gold substrate and the liquid metal. Figure 6 presents a comparison of the same line segment over the course of several days, with Fig. 6a taken directly after the pattern was formed. After 24 h (Fig. 6b), the spreading fronts on either side have exceeded the width of the original line, the outline of which is clearly visible. By that time, the line was coated by a passivating metal oxide layer, although the active reaction with the underlying gold, even after 7 days (Fig. 6c), suggests that liquid metal can still flow and diffuse into the gold region far beyond the contact area. Figure 6d–f show electron micrographs at higher resolution of the metal line and its surroundings, revealing the formation of several distinct crystallites of varying sizes.

We discovered that the diffusion of EGaIn on the gold layers depended on the thickness of the gold. By examining the reaction to the deposition of EGaIn on three substrates, with nominal gold thicknesses of 10 nm (Fig. S3), 40 nm (Fig. S4) and 100 nm (Figs. 5 and 6), we observed significant diffusion for 40 nm and 100 nm, and no diffusion for 10 nm. For the thicker layers (> 40 nm), the diffusion behavior of In differed from that of Ga in the Au layer. Gallium diffusion into the gold appears as chemical wave-like behavior (Fig. S4g), while Indium seems to slowly disintegrate into smaller particles (Fig. S4h). For the thin layer (10 nm), the absence of visible diffusion suggests that after the thin layer of gold directly below the EGaIn had been consumed, there was not enough gold to accommodate lateral diffusion to the sides.

We examined pattern formation for two more eutectic GaIn alloys on gold, GaInSn and GaInZn (see Supplementary Information, Fig. S5). GaInSn produced thicker lines, 2.5–3 times wider than EGaIn (Fig. S5a,b), whereas GaInZn did not seem to wet the gold even after treatment with HCl, producing large disconnected globules (Fig. S5c). This suggests that an oxide film forms more rapidly on GaInZn than on EGaIn, perhaps due to the oxidation propensity of metallic Zn^26^, thereby disrupting its chemical contact with the underlying gold surface. It is possible to devise methods to overcome this limitation, by controlling the environment of the liquid, either by depositing in an inert environment or by encapsulating the liquid metal directly during deposition. This should open up new venues for metal liquid nanocapsules^27, 28^.

## Conclusions

This work demonstrates the importance of controlling the balance between surface tension and surface adhesion in pattern formation of liquid metal at the micron range. In all the conducted experiments, with all the different liquid metals used in this study on either gold or silicon oxide substrates, one common feature was observed, namely that as the liquid metal was extruded through the pore, it expanded well beyond the dimensions of the pore. Furthermore, in the case of SiO_2_, there was no chemical reaction between the liquid metal with the underlying surface. As the metal was extruded, an oxide film began to form and the metal line consisted of connected blobs of metal, created by spontaneous droplet formation. In this case, we may distinguish between the dynamic deposition of the initial line, and the stable printed pattern that is formed spontaneously afterwards, with characteristic widths of 1–3 µm, and 10–15 µm, respectively. In the case of gold-coated SiO_2_, the alloying reactions—so-called *reactive wetting*—were sufficient to prevent droplet formation, and instead favored the formation of a continuous line, albeit wider due to diffusive spreading. The considerably wider pattern—more than two orders-of-magnitude greater than the size of the pore—could be attributed to the strong contact between the glass tip and the underlying substrate. This pattern, too, proved unstable for thick gold films (> 40 nm) due to reactive wetting which ultimately altered its shape. Nonetheless, it may still be possible to reduce the width by controlling the tip-substrate distance, and depositing the metal at a slightly elevated position above the substrate, whereas the extent of the reactivity could be controlled by depositing on thin gold films (~ 10 nm). By controlling both the distance to and the thickness of the gold film, we estimate that it would be possible to achieve stable patterns with sub-micron width on gold-coated substrates.

## Methods

Eutectic GaIn was purchased from Sigma Aldrich. Gallium Alloys, GaInZn (67:29:4%) and GaInSn (62:25:13%), were purchased from Kojundo Chemical Laboratory Co, Ltd., Japan. Solvents were purchased from Wako Pure Chemicals, Japan. Glass capillaries (with filament, Cat. No. GD-1) with a nominal O.D. of 1 mm, and length of 9 cm, were purchased from Narishige Co. Gold electroless plating was performed following the recipes of Jin et al.^24^ and Hu et al.^23^ with several modifications. For cleaning and surface hydroxylation, the glass tubes were immersed in a freshly made piranha solution (7:3::H_2_SO_4_:H_2_O_2_) for 30–60 min in a Teflon bath, rinsed thoroughly with triple-distilled water (TWD) and dried with a flow of N_2_ gas. For surface functionalization of the inner surface of the tubes, a solution of 9.1% APTMS in methanol was directly applied by connecting the capillaries to a syringe pump (either Harvard Apparatus Co., Model Pump 11 or YMC Co., Model YSP-201). The tubes were pumped 3–4 times, after which the free end was sealed with parafilm and left completely full overnight (16–24 h). The tubes were then cleaned by pumping methanol followed by TDW and dried with N_2_ gas. A solution of 10 nm Gold Nanoparticles (GNPs) in water (COSMO Bio Co., Cat. No. UG-10–20) was used to form the self-assembled monolayers of GNPs on the inner surface of the glass capillary, by pumping the dry functionalized tubes with the GNP solution 3–4 times, before sealing the free end and leaving it for 3–4 h. The electroless deposition was activated by introducing a solution of 0.5–1% Hydrogen tetrachloroaurate (III) hydrate (99.8%, STREM Chemical Inc.) and 5% H_2_O_2_ (50 wt%, Sigma-Aldrich Cat. No. 516813). The reaction proceeded as soon as the solution reached the cavity. After 20 min of slow pumping, the tubes were cleaned with TDW and dried with N_2_ gas. Water for all solutions was filtered through a 0.02 µm syringe filter (Whatman 6809-2102 Anotop 25). The quality of the long-term adhesion of the gold film to the glass was noted in two separate instances: one, in which several coated tubes were sonicated in TDW with the coating remaining intact, and the second time, when several tubes were left for 2–3 months inside a closed box, and the coating appeared intact.

P-type boron-doped silicon substrates with 300 nm oxide were purchased from the Electronics and Materials Co., Japan. Gold substrates were prepared by thermal vapor deposition (TVD) of Cr (~ 5 nm) followed by Au (~ 100 nm) on clean hydroxylated silicon substrates, producing a mean roughness of ~ 0.5 nm for smooth printing.

Two substrates were prepared for Energy Dispersive X-ray spectroscopy (EDS) measurements by TVD of Au(10 nm)/Cr(5 nm)/SiO_2_ and Au(40 nm)/Cr(5 nm)/SiO_2_. The EGaIn was patterned on these substrates by taking a very small portion of EGaIn and using ceramic tweezers to draw a line by gently and quickly sliding the tweezer on top of the Au substrate. All samples were stored in ambient conditions.

Pipettes were formed in a thermal puller, PC-100 (Narishige Co.), using one step at a nominal operating power of 61 W at full load. Prior to filling the pipette, diluted HCl was added to the liquid metal to remove the oxide. Excess HCl was then collected and removed. Initial filling of the pipette consisted of two steps: (1) a Hamilton syringe (#1705, 33 ga) introduced the metal liquid into the pipette; (2) the pipette was then placed inside a centrifuge (mini centrifuge, Model MF-12000, AS ONE Co.), for 90 s at 2000 rpm. The filled pipette was then placed inside a home-made motorized stage (Sigma Koki Co., Model SG SP 26-50) and pipette holder, controlled by PC with a dedicated LabVIEW program. Line patterning was monitored with a side camera (TOSHIBA, JK-TUS3H) and a top camera (SHODENSHA, CI500CU and HIROX, CX-7575CS), and was recorded for the duration of the experiment. Pressure was applied to the pipette using an HPLC pump, LC-10AD (Shimadzu Co.). Optical imaging of the substrate after deposition was performed using an Olympus BX51M microscope, operating in Bright Field or Dark Field modes. SEM imaging and EDS measurements were done in JSM-7600F (JOEL). EDS measurements were performed immediately (~ 1 h) after sample preparation and 1 week later. Acceleration voltage was 15 kV.

## Supplementary Information

Supplementary Video 1.

Supplementary Video 2.

Supplementary Video 3.

Supplementary Information 1.

## Data Availability

Supplementary Information is available from Nature website.
