# Supplementary material for: Sacrificial gold coating enhances transport of liquid metal in pressurized fountain pen lithography
Source: Sci Rep. 2021 Feb 25;11:4670. doi: 10.1038/s41598-021-84065-4 (PMC7907188; doi:10.1038/s41598-021-84065-4)
Supplement: Supplementary file 4 — Supplementary Information 1. [file 41598_2021_84065_MOESM4_ESM.pdf]

Supplementary Information

Sacrificial Gold Coating Enhances Transport of Liquid Metal in Pressurized Fountain Pen Lithography

Gideon I. Livshits, Jiannan Bao, Leo Sakamoto, Tomoki Misaka, Yuki Usami, Yoichi Otsuka, and Takuya Matsumoto

The Supplementary Information contains additional information: three videos and five figures, as referenced in the main manuscript.

Figure S1. Electroless plating of gold in practice.
Figure S2. Initial stage of printing EGaIn on SiO_2/Si.
Figure S3. SEM and EDS measurements of EGaIn on 10 nm gold-coated SiO2.
Figure S4. SEM and EDS measurements of EGaIn on 40 nm gold-coated SiO2.
Figure S5. GaIn-X, X= (Sn, Zn), on gold-coated silicon oxide.

Video S1
Video S1 is a video showing the complete filling of a nanopipette down to its pore in real time.

Video S2
Video S2 is a video showing (side and top views of) line deposition of EGaIn on SiO_2/Si in real time. The inset shows enlarged region (150%) of the area of deposition.

Video S3
Video S3 is a video showing (side and top views of) line deposition of EGaIn on Au/Cr/SiO_2 /Si in real time.

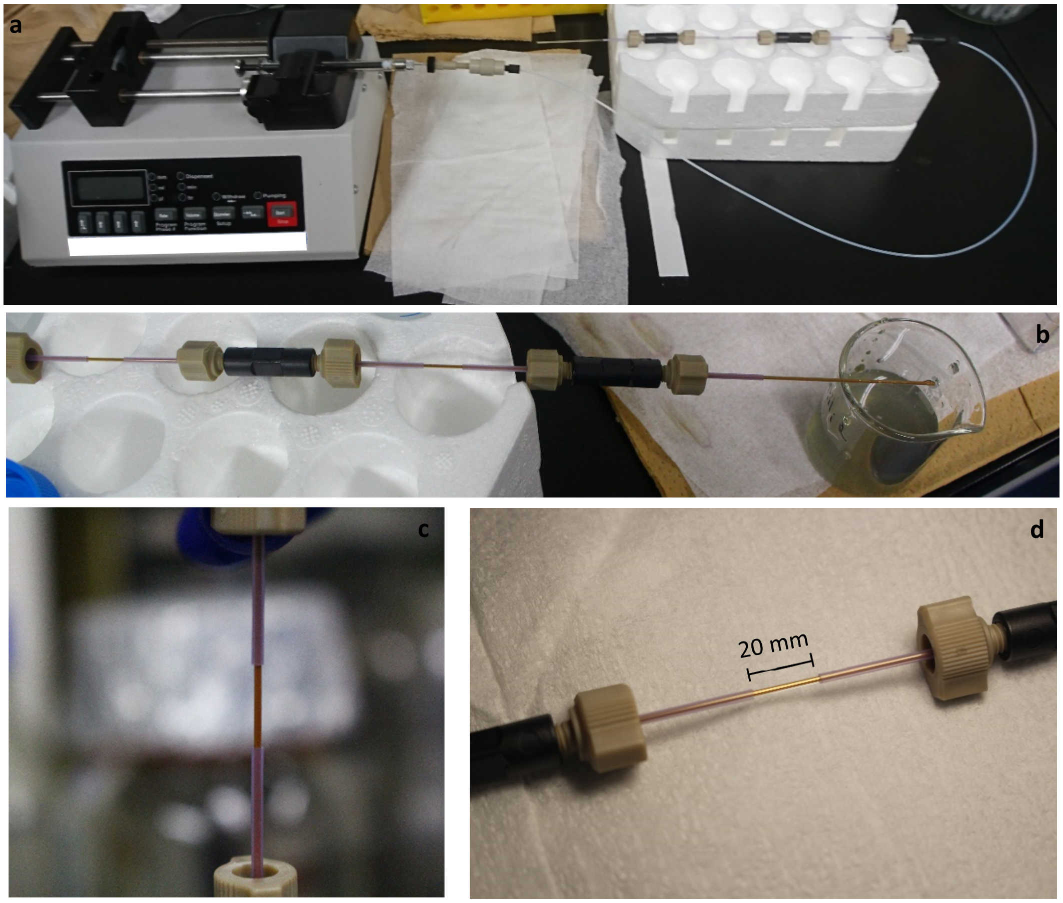

Figure S1. Electroless plating of gold in practice. (a) Photograph of three glass tubes (right), connected to each other and to a syringe pump (left). (b) A controlled flow is established for the reaction, and the liquid flows only through the tubes. (c-d) During the final stage of the synthesis, the tube turns a deep shade of red (c) and transforms into gold (d).

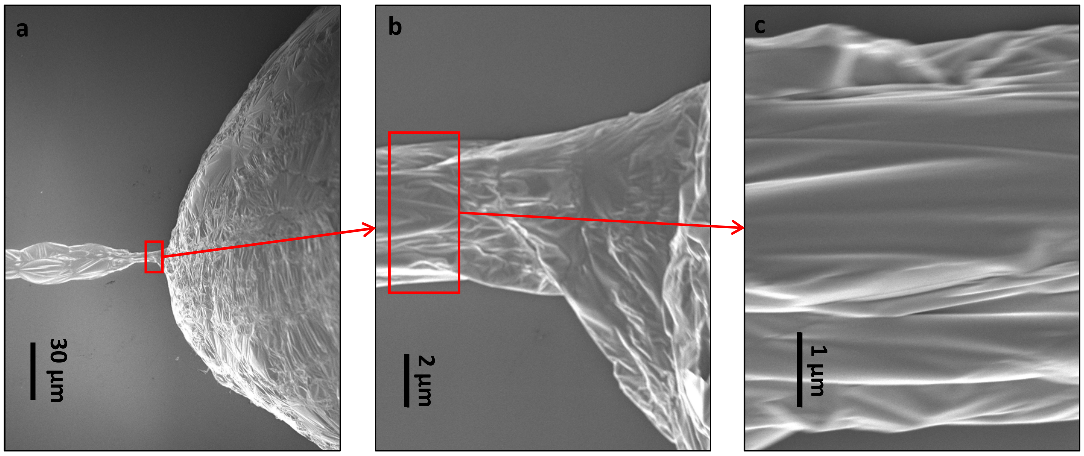

Figure S2. Initial stage of printing EGaIn on SiO_2/Si. (a)-(c) are a sequence of SEM images of increasing resolution near the initial point where the line is drawn. The completed pattern appears in Fig. 4 in the main manuscript. In (a), the front of the drop is clearly visible on the right. In (b), the position where the nanopipette emerges from the front is clearly visible, forming a cone. As the substrate is moved, the line begins to form, swelling up from its dynamic size, estimated to be of the order of 1 µm (as shown in Fig. 4c and Video S2). In (c), a narrow segment of the line is shown, revealing the complex surface geometry of the line. The width of the narrow segment is ~5 µm.

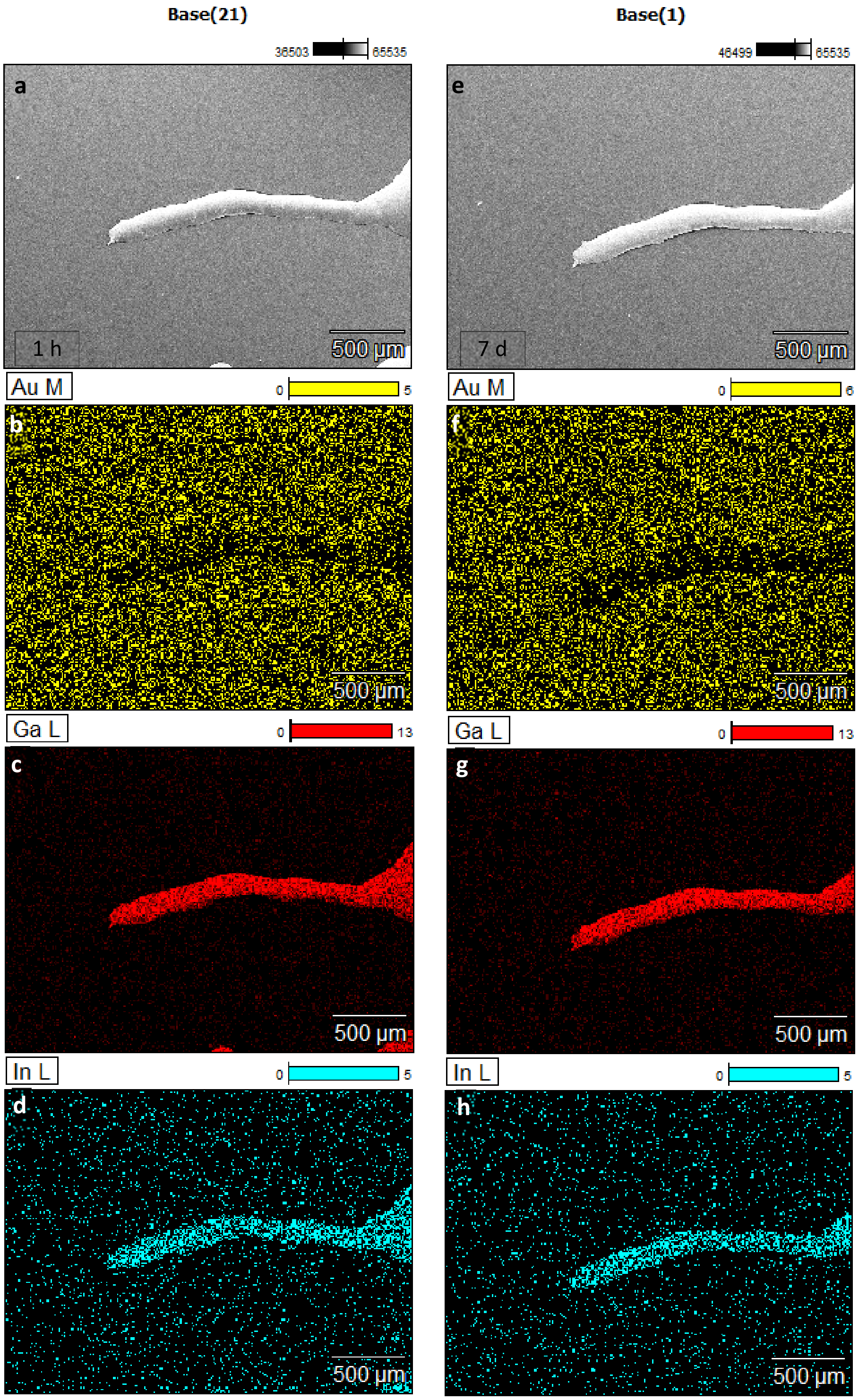

Figure S3. SEM/EDS measurements of EGaIn on 10 nm gold-coated SiO_2. (a)-(d) and (e)-(h) are SEM and EDS measurements taken 1 hour and 1 week, respectively, after sample preparation. (b), (f) EDS mapping of Au M-shell emission; (c), (g) EDS mapping of Ga L-shell emission; (d), (h) EDS mapping of In L-shell emission. Comparing these images, we observe little change around the pattern or inside the pattern. It seems that 10 nm of Au are not sufficient to produce lateral diffusion.

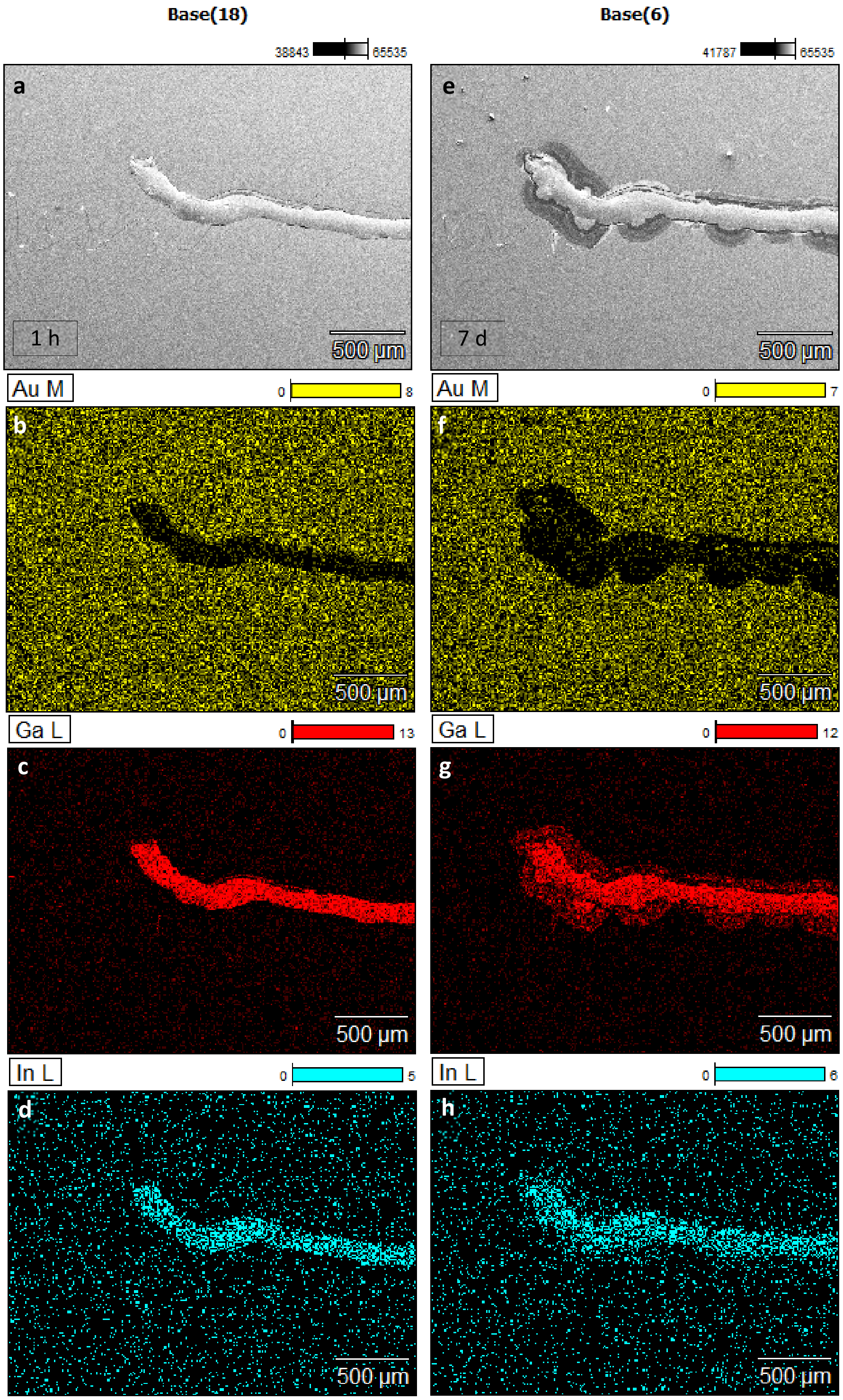

Figure S4. SEM/EDS measurements of EGaIn on 40 nm gold-coated SiO_2. (a)-(d) and (e)-(h) are SEM and EDS measurements taken 1 hour and 1 week, respectively, after sample preparation. (b), (f) EDS mapping of Au M-shell emission; (c), (g) EDS mapping of Ga L-shell emission; (d), (h) EDS mapping of In L-shell emission. The dark contrast region around the perimeter of the deposited pattern of EGaIn that could not be confirmed after 1 hour in (a) appears quite clearly after 1 week in (e). Comparing (b) and (f), we observe the signal intensity of Au decreases in the dark region. Whereas (c) and (d) show Ga and In are localized inside the boundary of the GaIn island, (g) and (h) reveal the presence of Ga and In beyond this boundary, inside the dark region. Note also the clear sign of In disintegration, when comparing (d) and (h). We conclude that EGaIn diffuses into the Au film.

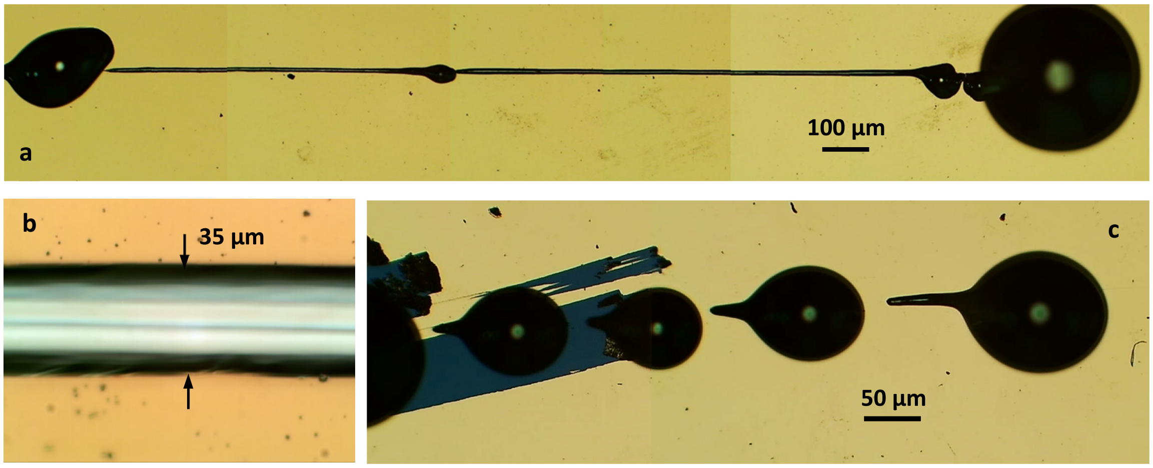

Figure S5. GaIn-X, X= (Sn, Zn), on gold-coated silicon oxide. (a) Successive optical images of a successful extrusion of GaInSn on gold (at ~70 Atm) taken directly after deposition. A long line was drawn, with one discontinuity. (b) A short segment of the line in (a) in greater resolution, reveals an average width of ~35 µm. (c) Extrusion of GaInZn on a gold coated SiO_2. As with all liquid metals in this study, GaInZn was treated with HCl prior to deposition, but it was not possible to obtain long continuous patterns, producing instead large globules on the surface. Part of the GaInZn was deposited on exposed SiO_2 (left side), with the similar pattern suggesting a passivating oxide film formed rapidly during the extrusion, preventing chemical adhesion to either surface.
